# Methods of high integrity RNA extraction from cell/agarose construct

**DOI:** 10.1186/s13104-015-1627-5

**Published:** 2015-11-04

**Authors:** Takahiro Ogura, Akihiro Tsuchiya, Tom Minas, Shuichi Mizuno

**Affiliations:** Department of Orthopedic Surgery, Brigham and Women’s Hospital, Harvard Medical School, 75 Francis St., Boston, MA 02115 USA; Funabashi Orthopaedic Hospital Sports Medicine Center, 1-833, Hasamacho, Funabashi, Chiba 274-0822 Japan

**Keywords:** Agarose gel, RNA, RNA integrity, Chondrocyte, Polysaccharide, Tissue engineering

## Abstract

**Background:**

Agarose hydrogels are widely used for three-dimensional cell scaffolding in tissue engineering and cell biology. Recently, molecular profiles have been obtained with extraction of a minimal volume of RNA using fluorescent-tagged quantitative polymerase chain reaction (qPCR), which requires high integrity RNA. However, the agarose interferes considerably with the quantity and quality of the extracted RNA. Moreover, little is known about RNA integrity when the RNA is extracted from cell/agarose construct. Thus, in order to obtain RNA of sufficient integrity, we examined various extraction methods and addressed reproducible methodologies for RNA extraction from cell/agarose constructs using spectrophotometry and microfluidic capillary electrophoresis.

**Results:**

With various extraction methods using a mono-phasic solution of phenol and guanidine isothiocyanate, we evaluated quantity and quality of total RNA from cell/agarose construct. Extraction with solution of phenol and guanidine isothiocyanate followed by a silica based membrane filter column gave sufficient RNA integrity number, which allowed us to proceed to fluorescent-tagged qPCR for evaluating various cellular activities.

**Conclusions:**

The RNA extraction methods using phenol and guanidine isothiocyanate solution and a silica membrane column can be useful for obtaining high integrity RNA from cell/agarose constructs rich in polysaccharide and extracellular matrix. Our study contributes to further investigation using agarose hydrogels and other materials rich in polysaccharide in the field of cellular and tissue engineering.

## Background

Agarose hydrogels have been conveniently used for three-dimensional (3-D) cell scaffolding. This is largely because the gels promote the maintenance of chondrogenic phenotypes, e.g., the synthesis of cartilaginous extracellular matrix (ECM), for neocartilage engineering and cell biology applications [1–3].

Cells embedded in the agarose are supported by the 3-D environment and homogeneously distributed, so that the gel is suitable for investigating cellular functions in response to biophysical stresses [4–6]. Recently, gene expression profiles have begun to provide useful information for analyzing the activities of various genes. To obtain those profiles, high quality RNA was required for the quantitative assaying of multiple genes. During RNA extraction from cell/agarose constructs, however, the agarose interfered considerably with the yield and purity of the RNA [7, 8]. A mono-phasic solution of phenol and guanidine isothiocyanate, TRIzol^®^ Reagent (Life Technology, CA, USA), has recently been used for RNA extraction from multiple organisms. After dissolving agarose gels with this solution and adding chloroform to separate the phases, subsequent RNA precipitation with isopropanol results in contamination of RNA with agarose. This contamination frequently hinders downstream applications, including gene expression analysis using fluorescent-tagged quantitative polymerase chain reaction (qPCR). Although the extraction methods were modified to reduce the agarose contamination [9, 10], information about RNA integrity has not been obtained. Thus, we evaluated the various extraction methods, including a commercially available kit, and determined the resulting RNA integrity using spectrophotometry and microfluidic capillary electrophoresis.

## Methods

### Cell isolation and seeding

Bovine forelimbs (from calves 2–3 weeks old) were purchased from a local abattoir. Full-depth articular cartilage pieces were harvested from the humeral condyle using a surgical blade (No. 15, BD, Franklin Lakes, NJ, USA). The harvested cartilage pieces were washed with Dulbecco’s phosphate-buffered saline (D-PBS, Life Technology, Carlsbad, CA, USA), minced, and digested with 0.2 % type I collagenase (CLS; Worthington Biochemical, Lakewood, NJ, USA) dissolved in Ham’s F-12 medium (Life Technology) with gentle rotating for 18 h at 37 °C. Bovine articular chondrocytes (bACs) were isolated from the digestion with a cell strainer (70 μm, BD) by removing non-digested debris, followed by rinsing twice with D-PBS and centrifugation at 220×*g* for 5 min.

**Fig. 1:**
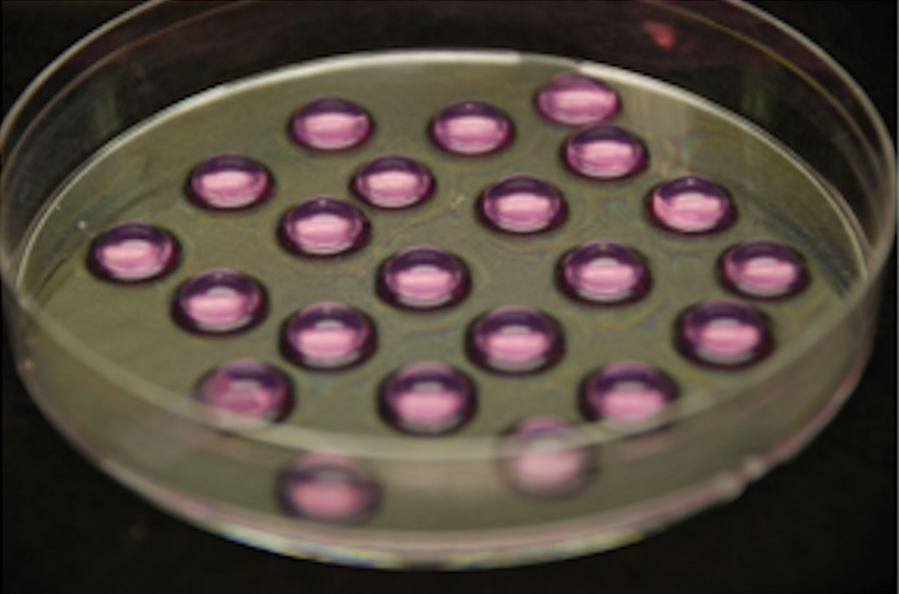
Cell/agarose constructs. Bovine chondrocytes suspended in 100 µl of agarose hydrogels were seeding onto a non-adherent 100-mm culture dish

For making cell/agarose constructs, 1.5 % culture-grade agarose (Sigma-Aldrich, St. Lois, MO, USA) dissolved in D-PBS was autoclaved at 120 °C for 15 min followed by maintaining at 37 °C. Five hundred thousands cells were suspended in 100 µl of the warm agarose at 37 °C. The cell/agarose suspension was poured on a non-adherent 100-mm culture dish (Fisher) and cooled to room temperature for agarose solidification (Fig. 1).

For the cell-only control, the same number of the cells were seeded to 12-well plates (4.5 cm^2^/well, Falcon). For the agarose control, the same amount of agarose without cells was poured on the non-adherent culture dish. The bAC/agarose constructs were incubated in Dulbecco’s modified Eagle’s medium (DMEM)/Ham’s F-12 (1:1) medium (Life Technology) including 10 % fetal bovine serum, 100 units/ml penicillin and 100 µg/ml streptomycin at 37 °C and 5 % CO_2_ in air for 4 days.

### Histological evaluation

Cell distribution and chondrocytic phenotypes within agarose hydrogels were evaluated histologically. A cell/agarose construct was harvested at day 4, fixed with 2 % paraformaldehyde (Fischer) in 0.1 M cacodylic acid (Polysciences, Warrington, PA, USA), and embedded in methacrylate resin (Technovit^®^ 7100, Heraeus Kulzer, Germany). To reveal the presence of sulfated glycosaminoglycan, a 10-µm section was stained with 0.5 % Toluidin blue-O at pH 4.0 (Fisher).

### Improvement of RNA extraction methods

**Fig. 2:**
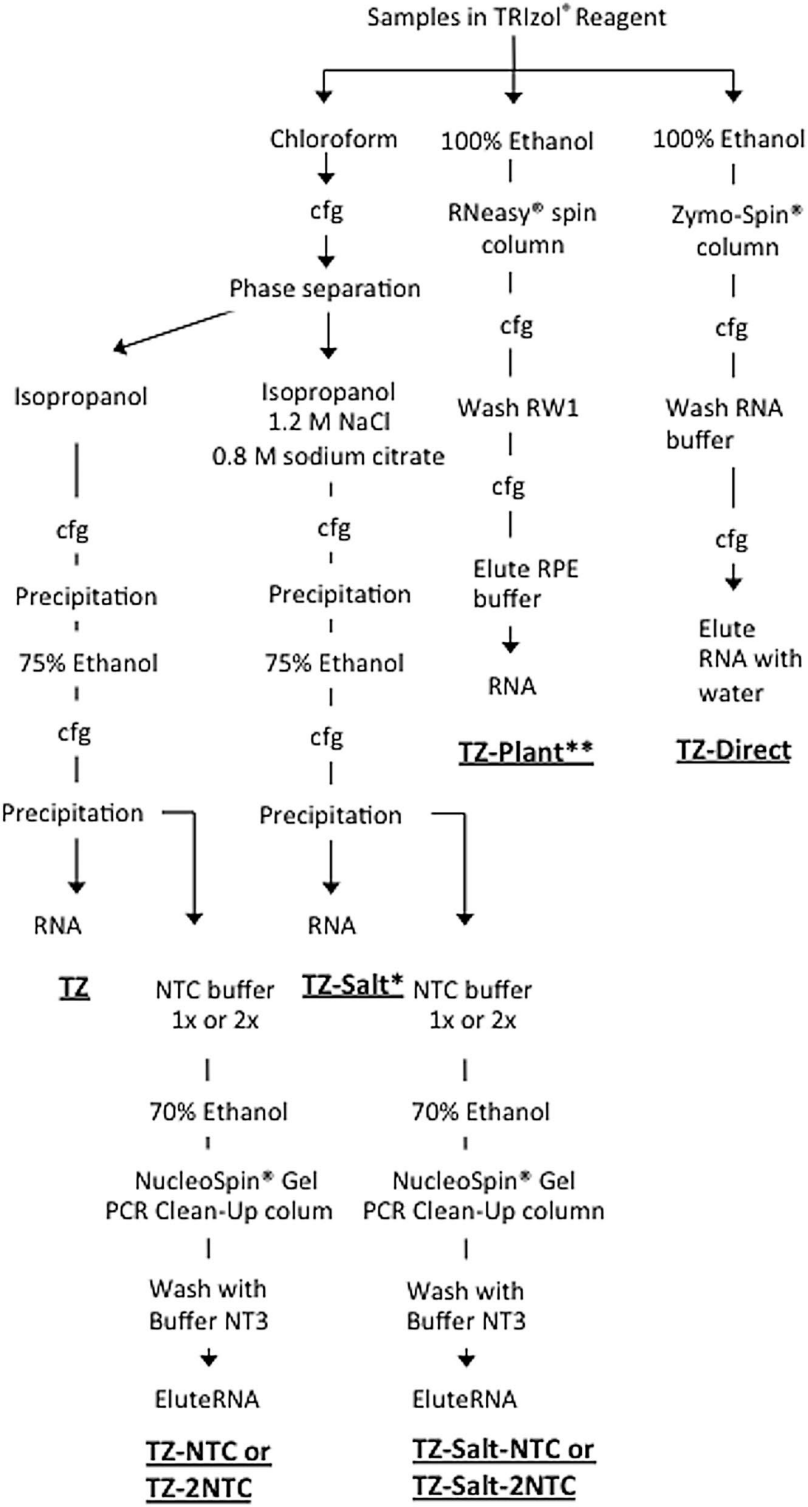
RNA extraction methods. *cfg* centrifugation

The cell/agarose constructs were harvested and immediately homogenized in 1-ml of phenol and guanidine isocyanate reagent (TRIzol^®^ Reagent, Life Technology) with a pellet pestle (Kimble-Chase^®^, Thermo-Fisher). We examined those homogenized samples with various RNA extraction methods as follows (Fig. 2):

1. Phase separation with chloroform by centrifugation, and precipitation with isopropanol by centrifugation, per manufacturer’s instructions (TZ).
2. After phase separation with chloroform, the aqueous phase was transferred to a separate tube. A half volume of high-salt solution composed of 1.2 M sodium chloride (NaCl) and 0.8 M sodium citrate and a half volume of isopropanol were then added, followed by centrifugation (TZ-Salt) [9, 11].
3. After TZ or TZ-Salt preparation and centrifugation, the precipitant was dissolved in 200 µl of potassium thiocyanate buffer (NTC buffer, prepared by a manufacturer, Macherey–Nagel, Duren, Germany) per 100 mg of agarose and incubated at 50 °C for 10 min. The sample in NTC buffer was mixed with an equal volume of 70 % ethanol, followed by centrifugation with a silica spin column (NucleoSpin^®^ Gel and PCR Clean-Up, Macherey–Nagel). The column binding RNA was washed with ethanol-based buffer (NT3 buffer, prepared by the manufacturer, Macherey–Nagel) and eluted with low ionic strength conditions using alkaline buffer (NE buffer, Macherey–Nagel; TZ-NTC, TZ-Salt-NTC).
4. When RNA was dissolved with NTC in the above TZ-NTC and TZ-Salt-NTC processes, the RNA was dissolved with 2× volumes of NTC (TZ-2NTC or TZ-Salt-2NTC).
5. One volume of sample homogenate in TRIzol^®^ Reagent was mixed with a half volume of 100 % ethanol, followed by centrifugation with a silica based membrane filter (RNeasy^®^ Mini Plant kit, Qiagen, Valencia, CA, USA). The RNA bound to the membrane filter was washed with guanidine salt and ethanol based buffer (RW1) and eluted with RPE buffer (not defined by manufacturer) following the manufacturer’s protocol (TZ-Plant) [10].
6. One volume of sample homogenate in TRIzol^®^ Reagent was mixed with one volume of 100 % ethanol followed by centrifugation with a silica based membrane filter (Direct-zol™ RNA kit, Zymo Research, Irvine, CA, USA) (TZ-Direct). The RNA bound to the membrane filter was washed with Direct-zol™ RNA PreWash and RNA Wash Buffer followed by elution with RNase-free water following the manufacturer’s protocol.

Extraction of RNA from bACs cultured in monolayer without agarose was conducted for a cell-only control. In addition, agarose was used for a cell-free control with TZ to determine the effects of agarose hydrogels.

### Concentration, purity, and integrity of the extracted RNA

We evaluated the concentration, purity, and integrity of RNA extracted with various extraction methods from the bAC/agarose hydrogel construct. We measured the optical density (OD) of 1.5-µl of extracted RNA at 230, 260 and 280 nm with a spectrophotometer (NanoDrop ND-1000, Thermo Scientific, DE). The RNA concentration was converted from OD at 260 nm, and the purity was evaluated by the OD ratio at 260/280 nm for protein contamination and the OD ratio at 260/230 nm for polysaccharide, phenol and other contaminations. We also measured the concentration and the integrity of RNA with a fluorescent-tag and microfluidic capillary electrophoresis (Bioanalyzer^®^, Agilent Technologies, CA, USA). RNA was separated in the channels of the microfabricated chip (Agilent) due to molecular weight and detected with a fluorescence detector. RNA integrity was expressed as the RNA integrity number (RIN, Agilent 2100 expert software, Agilent Technologies, CA, USA). RIN values ranged from 10 (intact) to 1 (totally degraded), with an RIN higher than 8.0 indicative of high integrity [12].

### Data analyses

Three samples were evaluated for each method. The volume of final elution buffer was 30 µl in all methods except the TZ-Direct method, which was 50 µl. Corrected concentrations of TZ-Direct extracted RNA were used. The mean and standard deviation (SD) were calculated for all measurements.

## Results

**Fig. 3:**
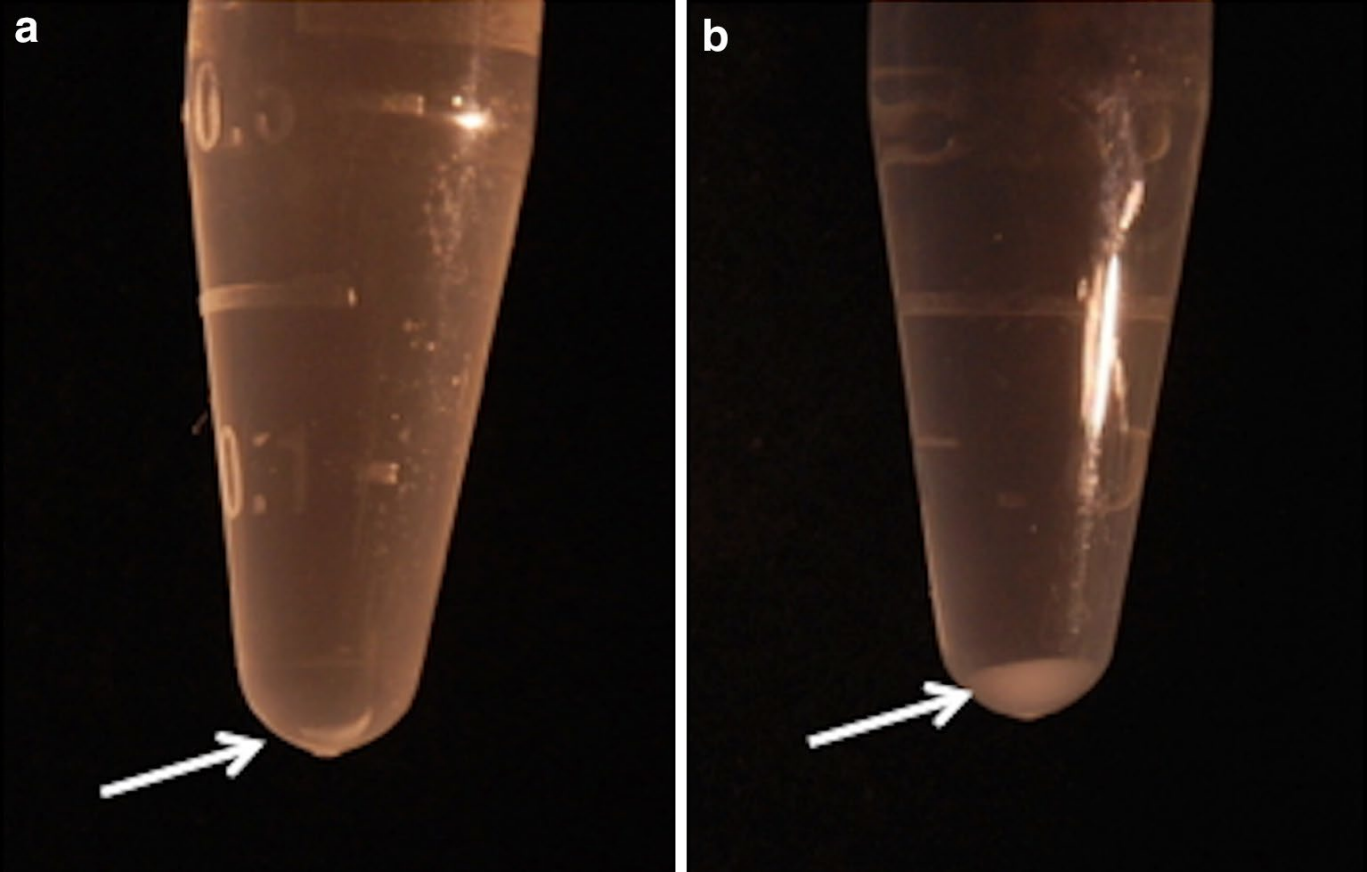
RNA precipitation with isopropanol using TRIzol^®^ Reagent. Precipitation (*arrow*) from cell-only control in 1.5 ml tube (**a**), cell/agarose construct (**b**). The amount of RNA precipitation (**b**) was larger than that from the cell-only control (**a**)

The final precipitations of RNA with isopropanol in both the TZ and TZ-Salt groups were gel-like stiff, large and glossy compared to the ones in the cell-only control group (Fig. 3). This precipitation was not dissolved in water by pipetting.

### Histological evaluation

**Fig. 4:**
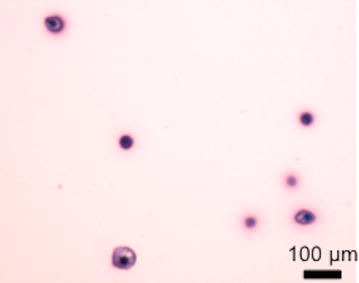
Bovine articular chondrocytes in agarose hydrogel. A 10-µm section was stained with Toluidine blue-O. Metachromatic matrix indicated cartilage ECM

bACs were distributed homogeneously throughout an agarose hydrogel (Fig. 4).

The bACs produced a metachromatic matrix around the cells, representing cartilage ECM. Picnotic cells were not seen, indicating that the bACs maintained their biosynthetic capabilities within the agarose hydrogel constructs.

### Concentrations

**Fig. 5:**
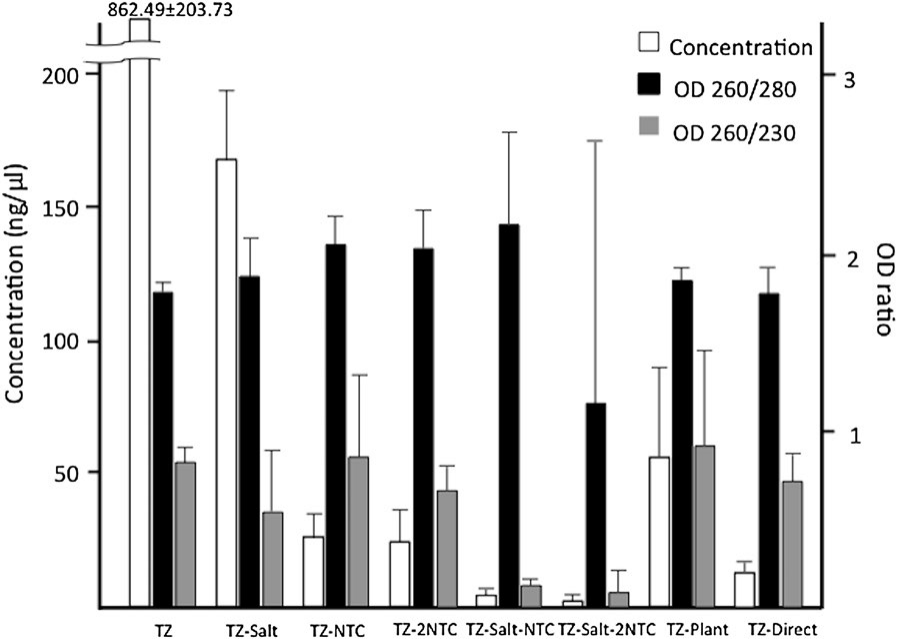
Concentration (ng/µl), ratio of OD 260/280 nm and OD 260/230 nm by spectrophotometry. In the cell-only control, concentration, OD 260/280 nm and OD 260/230 nm were 263.47 ± 49.2 (ng/µl), 1.93 ± 0.06 and 0.56 ± 0.12, respectively. The *bars* stand for the standard deviations (n = 3)

**Fig. 6:**
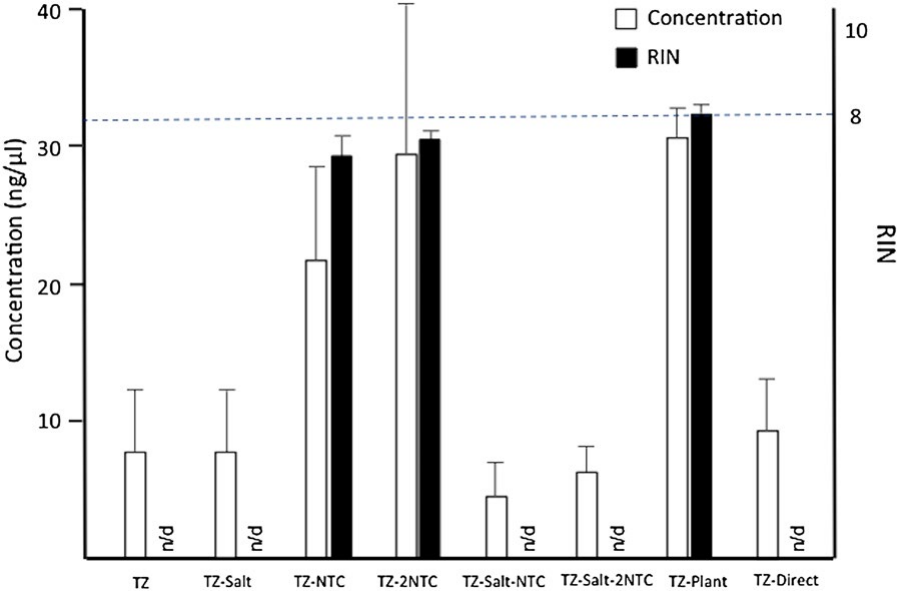
Concentration (ng/µl) and RIN by microfluidic capillary electrophoresis. In the cell-only control, the concentration and RIN were 67.33 ± 21.39 (ng/µl) and 8.8 ± 1.04, respectively. The *bars* stand for the standard deviations (n = 3). *n*/*d* not detected

The concentrations were measured by a spectrophotometer and fluorescent tag microfluidic capillary electrophoresis. The RNA concentration extracted from the cell-only control was 263.47 ± 49.20 and 87.33 ± 21.39 (ng/µl) by spectrophotometry and microfluidic capillary electrophoresis, respectively. The RNA concentration was determined by OD at 260 nm, which can be compromised by the presence of DNA and phenol [13]. One of the possibilities for the higher concentration in spectrophotometry was that the phenol from TRIzol^®^ Reagent could interfere with OD 260 nm. A higher level of extracted RNA was seen by spectrophotometry compared to microfluidic capillary electrophoresis, which was also seen in TZ and TZ-Salt. Moreover, in the TZ and TZ-Salt groups, the concentrations by spectrophotometry were higher than cell-only control, whereas the concentrations by microfluidic capillary electrophoresis were lower than the cell-only control. In TZ-NTC and TZ-2NTC, the concentrations were similar by both spectrophotometry and microfluidic capillary electrophoresis. By adding salt to TZ-NTC and TZ-2NTC, the concentrations in TZ-Salt-NTC and TZ-Salt-2NTC showed lower than without salt by spectrophotometry and microfluidic capillary electrophoresis. Using the Plant kit, the concentrations by spectrophotometry showed a higher trend compare to by microfluidic capillary electrophoresis. Using the Direct-zol kit, the concentrations showed a decreased trend by both spectrophotometry and microfluidic capillary electrophoresis (Figs. 5, 6).

### Purity

The ratio of OD 260/280 nm and OD 260/230 nm were 1.93 ± 0.06 and 0.56 ± 0.12 in the cell-only control, respectively. The mean OD 260/280 showed a higher trend (>1.8) in the TZ-Salt, TZ-NTC, TZ-2NTC, TZ-Salt-NTC and TZ-Plant methods. The mean OD 260/230 showed a lower trend in all methods, though the TZ-NTC and TZ-Plant methods produced the best values (Fig. 5).

### Integrity

**Fig. 7:**
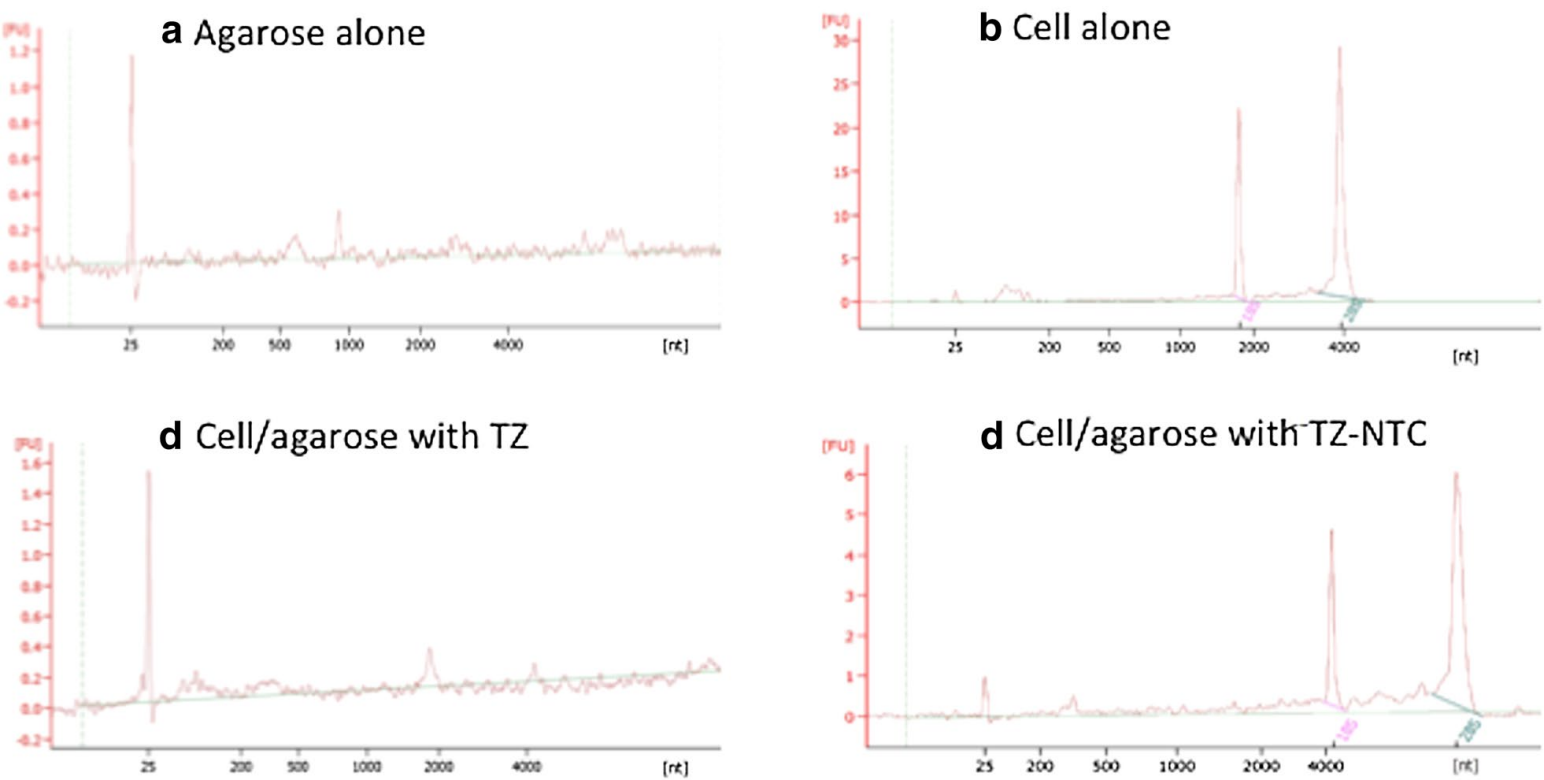
Chromatograms of microcapillary electrophoresis. Agarose-only (**a**), cell-only control by TRIzol^®^ reagent (**b**), cell/agarose construct by TRIzol^®^ reagent (**c**) and cell/agarose construct by TRIzol^®^ reagent combined with NucleoSpin^®^ Gel and PCR clean-up (**d**). *FU* fluorescent unit, *nt* nucleotide

The RIN of the cell-only control extracted with TZ was 8.8 ± 1.0. This indicated a high quality result for a fluorescent-based assay. Conversely, the RIN of cell/agarose constructs extracted with TZ and TZ-Salt was unable to produce any detectable values. However, by using NTC buffer, the RIN of cell/agarose constructs with TZ-NTC and TZ-2NTC were 7.3 ± 0.35 and 7.6 ± 0.15, respectively. In contrast, the RIN in TZ-Salt-NTC and TZ-Salt-2NTC was undetectable. In the TZ-Plant method, the RIN was 8.1 ± 0.15. In the Direct method, the RIN was 2.5 in one of our three samples, with the rest being undetectable (Fig. 6; Table 1). On electrophoresis, the agarose-only no-cell control showed a fluctuating baseline and no peak, and the cell-only control showed a typical electropherogram of high-quality RNA, including a clearly visible 28S:18S rRNA peak. TZ extraction method for cell/agarose constructs showed a fluctuating baseline and no peak, which was similar to agarose-only no-cell control. TZ-NTC and TZ-2NTC extraction methods showed a peak type similar to the cell-only control, which indicated high-quality RNA and successful isolation of RNA from the cell/agarose constructs (Fig. 7; Table 1). In addition, the TZ-Plant method electropherogram showed a peak similar to the TZ-NTC and TZ-2NTC methods (data not shown).

**Table 1:** Concentration, purity and integrity of extracted RNA from different methods

	Concentration (ng/µl)		Purity		Integrity
	Spectrophotometer	Microfluidic capillary electrophoresis	OD 260/280	OD 260/230	RIN
TZ (control)	263.47 ± 49.2	67.33 ± 21.39	1.93 ± 0.06	0.56 ± 0.12	8.8 ± 1.04
TZ	862.49 ± 203.73	8.67 ± 4.93	1.77 ± 0.05	0.32 ± 0.085	n/d
TZ-Salt	167.46 ± 26.49	8.67 ± 4.93	1.86 ± 0.21	0.54 ± 0.34	n/d
TZ-NTC	26.10 ± 9.03	24.33 ± 7.51	2.04 ± 0.17	0.85 ± 0.46	7.3 ± 0.35
TZ-2NTC	25.04 ± 11.98	33 ± 12.17	2.02 ± 0.21	0.66 ± 0.13	7.6 ± 0.15
TZ-Salt-NTC	4.55 ± 2.00	5 ± 2.65	2.15 ± 0.51	0.123 ± 0.032	n/d
TZ-Salt-2NTC	2.45 ± 2.51	7 ± 2	1.15 ± 1.48	0.09 ± 0.12	n/d
TZ-Plant	55.96 ± 33.54	34.33 ± 2.31	1.84 ± 0.07	0.91 ± 0.53	8.1 ± 0.15
TZ-Direct	13.05 ± 5.71	10.56 ± 6.94	1.53 ± 0.14	0.71 ± 0.15	n/d

Date presents mean ± standard deviation

*n*/*d* not detectable, *RIN* RNA integrity number, *TZ* TRIzol^®^ reagent, *NTC* NucleoSpin^®^ Gel and PCR clean-up, *Plant* RNeasy^®^ Mini Plant kit, *Direct* Direct-zol RNA kit

## Discussion

Agarose hydrogels have been used for constructing 3-D chondrocyte cultures, as they allow the cells to efficiently accumulate newly synthesized cartilage matrix and maintain their phenotypes for a longer duration in culture [14]. The chondrocytes also resume their phenotypes in agarose gel even after multiple passages in a monolayer culture [1]. Thus, agarose is an advantageous biomaterial, particularly because of its biocompatibility for engineering a cell construct. It is a natural polysaccharide made of alternating β-d-galactpyranosyl monomers units and 3, 6-anhydro-l-galactpyranosyl monomer units [15]. Since agarose is a well-defined biomaterial, applications utilizing it should be explored in cell biology and tissue engineering. However, its residual in the RNA extract limits extending applications. We motivated to extract a higher integrity of RNA in order to measure multiple gene expressions and therefore understand and reproduce biological events. Collagen and gelatin are also widely used for a 3-D culture [16–21]. Those raw materials are mainly extracted from connective tissue in organisms. Thus, most available RNA extraction methods are already assured for qPCR. However, each cell type expresses unique behavior with material. Thus, agarose is still useful option for cell-based applications.

Phenol-based reagents, e.g., TRIzol^®^ Reagent, have been conveniently used for RNA extraction, which has been an improvement from the single-step RNA isolation developed by Chomczynski and Sacchi [22]. During cell homogenization without agarose, TRIzol^®^ Reagent efficiently maintains the integrity of RNA. Thus, we used the TRIzol Reagent for our studies. When this reagent was used for RNA extraction from cell/agarose constructs, the amount of RNA precipitation was larger than that from the cell-only control, indicating contamination of the agarose. Moreover, the concentration of the RNA was unrealistically high in comparison to the cell-only control, although the OD ratio at 260/280 nm indicated acceptable purity. In addition, the RIN of those samples was not detected due to a fluctuating profile of fluorescence units by microfluidic capillary electrophoresis. Thus, this discrepancy among measurements using TRIzol suggested agarose contamination. The polysaccharide in agarose was able to physically entrap the RNA and move it to the discarded phase during centrifugation, which resulted in low yield [23]. In addition, RNA precipitation with small fragments of polysaccharides resulted in inconsistent and low quality RNA [24, 25]. Many investigative methods, especially a sophisticated reverse transcription-qPCR, require a high quality of RNA, as expressed by RIN [26–28]. Therefore, we intended to improve the extraction methods to reduce agarose contamination.

Firstly we tried to avoid precipitation of the polysaccharide. We examined TZ-Salt to increase the salt concentration of precipitation with isopropanol by adding 1.2 M NaCl and 0.8 M sodium citrate. The amount of this precipitation was less than that with TZ, indicating that the polysaccharide contamination was successfully reduced. However, the RIN was not detected, although the OD ratio expressing purity was sufficient, as previously reported [9]. Thus, we added an additional step, using a silica membrane filter.

Secondly, RNA recovery from agarose gel after electrophoresis has been performed with NTC buffer and a silica membrane kit (NucleoSpin^®^ Gel and PCR Clean-Up kit), so we were motivated to use the same kit. The commercially available NTC buffer, which includes potassium thiocyanate, has been used for purification of DNA and RNA fragments from agarose gels. We used it for isolation of RNA from the agarose-contaminated precipitation with isopropanol. In TZ-NTC, the concentration and RIN were successfully obtained by microfluidic capillary electrophoresis. Our results showed that TZ-NTC was a reliable method for RNA extraction from cell/agarose constructs. For additional improvement, we attempted to use 2× volume of NTC to completely dissolve the precipitation instead of 1× volume of NTC buffer for TZ-NTC and TZ-Salt-NTC. However, there was no visible effect produced by 2× volume of NTC.

The RNeasy^®^ Mini Plant kit was designed for extracting RNA from plant tissue. The similarities between our agarose gel and polysaccharide rich plants motivated us to incorporate this Plant kit as well. Recently, Wang and his colleagues extracted RNA from human mesenchymal stem cells and murine chondrocytes embedded in agarose gel using this kit, but a RIN was not reported [10]. In addition, our samples were bACs embedded in agarose gel and cultured for 4 days. Since chondrocytes are known to accumulate a rich ECM (polysaccharide, etc.) under the conditions of 3-D culture, we evaluated whether the accumulated ECM interfered with TZ-Plant. We successfully measured an acceptable RIN with TZ-Plant method. This plant kit allowed the samples to centrifuge with TRIzol and its half volume of ethanol. This method was advantageous because it eliminated several steps before RNA elution. Overall, a silica gel membrane and ethanol were essential components for obtaining high yield and high integrity RNA. However, though Direct-zol was designed with a silica membrane, it was not useful. The manufacturer’s information was limited to clarifying the underlying mechanism of RNA selectivity.

## Conclusion

The TZ-NTC and TZ-Plant methods can be used to extract high integrity RNA from cell/agarose constructs rich in polysaccharide and ECM for fluorescent-tagged qPCR. Our findings contribute to a further investigation using agarose hydrogels and other materials rich in polysaccharide in the field of tissue engineering.

## Abbreviations

3-D: three-dimensional
qPCR: quantitative polymerase chain reaction
ECM: extracellular matrix
D-PBS: Dulbecco’s phosphate-buffered saline
bACs: bovine articular chondrocytes
DMEM: Dulbecco’s modified Eagle’s medium
OD: optical density
RIN: RNA integrity number
SD: standard deviation
